# Comparison of One- and Two-Photon Photoluminescence of Solution-Grown CsPbBr_3_ Bulk Crystals

**DOI:** 10.3390/ma19071303

**Published:** 2026-03-25

**Authors:** Da-Chuan Li, Zheng-Da Dong, Hou Wang, Yang Zhang, Chuan-Xiang Sheng

**Affiliations:** 1 State Key Laboratory of Photovoltaic Science and Technology, Department of Optical Science and Engineering, School of Information Science and Technology, Fudan University, Shanghai 200433, China; 23210720098@m.fudan.edu.cn (D.-C.L.); 23210720092@m.fudan.edu.cn (Z.-D.D.); 24210720013@m.fudan.edu.cn (H.W.); 2 School of Electronic Information Engineering, Guangdong University of Petrochemical Technology, Maoming 525000, China

**Keywords:** CsPbBr_3_ bulk crystal, excitonic luminescence, two-photon excitation, electron–phonon interaction

## Abstract

**Highlights:**

**What are the main findings?**

- Under 405 nm CW excitation, three distinct emissions—free excitons, exciton–LO–phonon sidebands with band tail effect, and trapped excitons—coexist; under 800 nm fs excitation, only trapped-exciton emission is observed.
- Temperature-dependent FWHM fitting yields an exciton–phonon coupling strength of ~85 meV for trapped excitons, markedly exceeding the ~45 meV found for free excitons.
- Temperature-dependent bandgap renormalization analysis gives an electron–phonon interaction (A_EP_) of 156 meV for trapped excitons versus 36 meV for free excitons.

**What are the implications of the main findings?**

- Two-photon excitation exhibits selective sensitivity to trapped excitons.
- Localization enhanced the electron–phonon coupling.
- Two-photon excitation PL would serve as a defect probe method.

**Abstract:**

We present a temperature-dependent photoluminescence (PL) study of solution-grown CsPbBr_3_ bulk crystal and thin film, using one-photon and two-photon excitations. Twin planes are observed in X-ray diffraction spectra in crystal. In analyzing PL peak position and spectral widths as function of temperature, we find that the electron–phonon interaction is generally stronger in CsPbBr_3_ crystals than in films. Moreover, with one photon excitation, emissions from excitons and trapped excitons are observed in CsPbBr_3_ crystal. Under two-photon excitation, only the emissions from trapped excitons are observed in bulk crystal. Our work demonstrates that two-photon excitation PL is more sensitive to the trapped excitons inside CsPbBr_3_, implicating an optical method to probe the inside quality of the crystal.

## 1. Introduction

Organic–inorganic halide perovskites have attracted broad research interest as promising materials for solar cells [1], light-emitting diodes (LEDs) [2,3,4], lasing [5], and photodetectors for the past decade [6,7]. Recently, all inorganic cesium lead halide perovskite (CsPbX_3_, X = Cl, Br, I) with enhanced emission and better stability has been reported extensively [8,9,10]. Moreover, CsPbBr_3_ bulk crystals, which could be grown in millimeter size using solution procession, had been proved to be among the promising candidates for X-ray and gamma-ray detection [11,12]. Recently, all inorganic perovskite CsPbBr_3_ nanocrystals exhibit enhanced two-photon absorption cross-section with increasing size, which could be orders of magnitude higher than CdSe and CdTe nanoparticles [13]. Two-photon pumped lasing based on CsPbBr_3_ crystals was also reported [14,15] as a promising candidate for possible application in multiphoton spectroscopic and frequency up-conversions.

Normally, in nanoparticles including CsPbBr_3_ [15], as well as CdSe, the PL spectra from two-photon absorption (TPA) excitation and one-photon absorption (OPA) excitation are similar, sometimes with slight red shift in TPA excited PL spectra, which has been attributed to size inhomogeneity and re-absorption [16,17], as well as to the effect of surface/defect states [18]. However, in CsPbBr_3_ bulk crystals (BCs) with ~millimeters size, the PL spectra of TPA and OPA are quite different; for example, the OPA PL could be multi-peaked [19], while the TPA PL usually contains less PL features [14]. Therefore, the optical properties of CsPbBr_3_ BC and its indication to optoelectronic application have not been fully addressed yet.

In this work, using solution procession, we obtain ~ millimeters size bulk crystal of CsPbBr_3_. Excited using 405 nm CW laser, we observed PL features from a few of sources including exciton, the trapped excitons et al. After using an 800 nm fs pulsed laser, only trapped excitons’ emissions were observed. We observed that the trapped excitons involved stronger electron–phonon interactions than excitons, which could be due to the complicated crystal structures in solution-processed bulk crystals.

## 2. Materials and Methods

### 2.1. Materials

Cesium bromide (CsBr, ≥99.5%), lead bromide (PbBr_2_, ≥99.5%), and choline bromide (CB, ≥98%) were purchased from Macklin (Shanghai, China).

CsPbBr_3_ bulk crystals are grown by the seed-crystal method. Fresh supersaturated precursor solution is prepared at room temperature by dissolving 4.8 mmol CsBr, 9.6 mmol ${PbBr}_{2}$, and 0.5 mmol CB in 5.3 mL DMSO solution completely. Then, 10 mL precursor solution is taken into a 20 mL bottle, and the sealed bottle is placed in dimethylsilicone oil at 90 °C. Millimeter-level CsPbBr_3_ seed-crystals can be obtained a few hours later. One CsPbBr_3_ seed-crystal is picked up as a seed and transferred into another 20 mL bottle with 10 mL precursor solution, and the sealed bottle is then placed in bath oil at 80 °C for 24 h, with the temperature gradually increased by 5 °C every 24 h. When the side length of the CsPbBr_3_ bulk crystal reaches more than 4 mm, the bulk crystal is picked out and cleaned with anhydrous ethanol.

The CsPbBr_3_ film was fabricated by the multi-step spin-coating method [20,21]. The cleaned glass substrate and the DMF solution of 1 mol/L ${PbBr}_{2}$ were preheated at 100 °C. The ${PbBr}_{2}$ solution was spin-coated on the substrate at 2000 rpm for 30 s and then dried at 100 °C for 30 min. After that, 0.07 mol/L CsBr methanol solution was spin-coated onto ${PbBr}_{2}$ film at 2000 rpm for 30 s and heated at 250 °C for 5 min. This process was repeated for seven to nine times until a high-quality CsPbBr_3_ film was obtained.

### 2.2. Methods

The photoluminescence (PL) spectrum was obtained by home-built systems, and the sample was put in a liquid nitrogen-cooled cryostat, in which the temperature could vary from 80 K to 300 K. The light source is either a continuous-wave (CW) diode laser at λ = 405 nm or fs pulsed laser at 800 nm. The fs pulsed laser has a high repetition rate (82 MHz) and low power (~0.2 nJ of energy/pulse). Both of the two laser spot diameters are approximately 1 mm. CW laser is slightly focused on the film with excitation intensity around 100 mW/cm^2^. The pulsed laser is focused using a convex lens with a focus length of 15 cm. The PL spectra were collected with a PG2000-Pro spectrometer (ideaoptics, Shanghai, China) in the backscattering geometry to minimize the influence of self-absorption. X-ray diffraction (XRD) was measured using a Bruker AXS Dimension D8 X-ray System (Bruker AXS, Karlsruhe, Germany). Time-resolved photoluminescence (TRPL) measurements were performed using a Pico-1000 time-correlated single-photon counting (TCSPC) system (Beijing Zhongke Kaichuang Technology Co., Ltd., Beijing, China) with ~100 ps temporal resolution. A picosecond pulsed laser (405 nm) was used as the excitation source.

## 3. Results and Discussion

**Figure 1:**
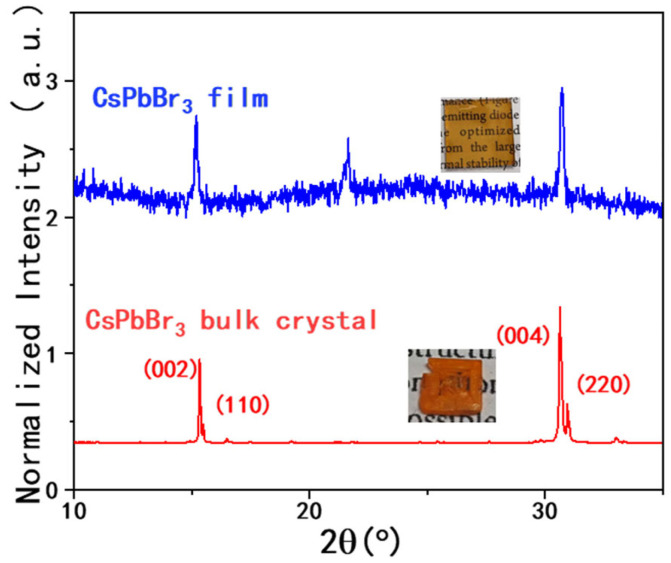
XRD patterns of CsPbBr_3_ thin film (blue) and CsPbBr_3_ bulk crystal (red).

Figure 1 presents XRD patterns of CsPbBr_3_ bulk crystal and film. A detailed description of materials and characterizations is included as the Experimental section in the Supporting Information. For film, the peaks of 2θ = 15.2°, 21.6°, and 30.5° indicate the solution processed CsPbBr_3_ perovskite film belonging to the pseudo-cubic phase at room temperature [22,23]. On the other hand, the XRD pattern of the orthorhombic CsPbBr_3_ bulk crystal presents the (004) and (220) peaks together, suggesting the existence of twin planes [24,25]. A detailed discussion is included in the Supporting Information in the section on XRD patterns. Nevertheless, the twin planes may be associated with the defects and spatial non-uniformity of the crystals.

**Figure 2:**
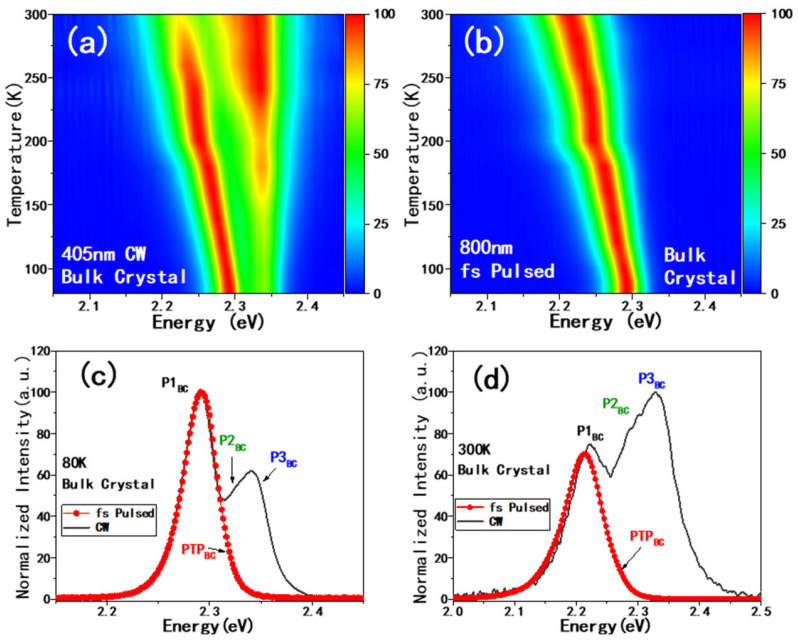
Temperature-dependent photoluminescence spectra of CsPbBr_3_ bulk crystal particles excited by a 405 nm CW laser (**a**) and 800 nm fs pulsed laser (**b**). (**c**,**d**) Comparison of PL spectra excited by CW laser and fs pulsed laser at 80 K and 300 K, respectively. The peak assignments are shown in Table S1 of Supplementary Information.

In Figure 2a,b, we included the normalized PL spectra of CsPbBr_3_ bulk crystals (BCs) at various temperatures plotted with false colors, excited by a 405 nm continuous wave (CW) laser (2a) and an 800 nm fs pulsed laser (2b), respectively. The spectra without normalization are also included as Figure S2 in Supplementary Information. Figure 2a presents two separate patterns for CW laser excitation; the higher-energy one actually can be divided as P2_BC_ and P3_BC_ (as shown in Figure 2c,d). P3_BC_ is at 2.35 eV (528 nm), which could be ascribed to excitonic emission [26,27], as generally observed in CsPbBr_3_ crystal or film.

**Figure 3:**
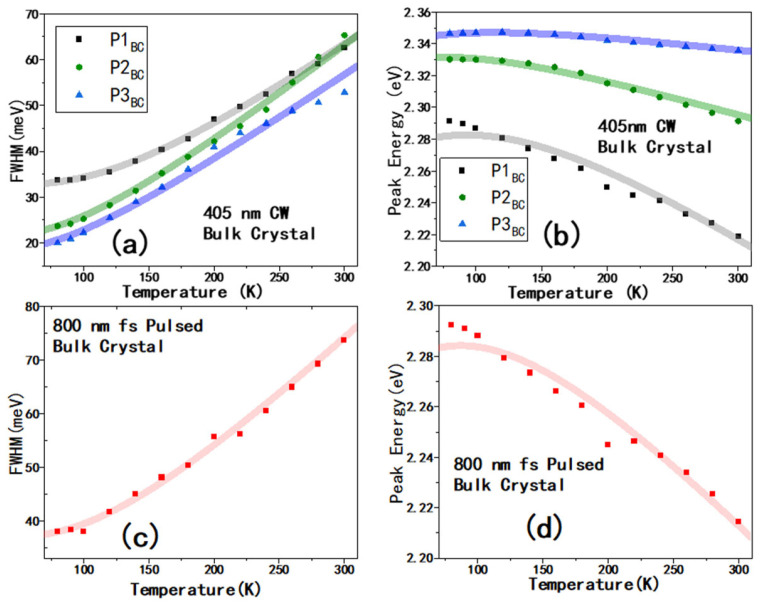
For a bulk crystal of CsPbBr_3_, (**a**,**b**) are PL FWHMs and peak positions of P1_BC_, P2_BC_, and P3_BC_ at various temperatures excited by a 405 nm CW laser, respectively; (**c**,**d**) are PL FWHM and peak position of PTP_BC_ at various temperatures excited by an fs pulsed laser at 800 nm. The peak assignments are shown in Table S1 of Supplementary Information.

Figure S3 of the Supplementary Information shows the typical PL spectra, along with fittings using three Lorentz functions for PL at 80 K and 300 K. Peak positions of P1_BC_, P2_BC_, and P3_BC_ excited by 405 nm as the function of temperature are summarized in Figure 3a. For both P3_BC_ and P2_BC_ emission, below 100 K, the peak energy is almost constant; above 100 K, the peak energy redshifts with increasing temperature. The energy difference between P2_BC_ and P3_BC_ is about 16 meV and 44 meV according to the fitting of asymmetric spectral shape, at 80 K and 300 K, respectively. We noticed from the literature that the Raman peak frequencies of CsPbBr_3_ are 71 cm^–1^ (8.8 meV), 91 cm^–1^ (11.3 meV), 128 cm^–1^ (15.8 meV), and 150 cm^–1^ (18.5 meV) [28]. Those phonon modes could contribute to the optical processes presenting as a phonon sideband with an averaged phonon energy [29]. However, P2_BC_ cannot be simply assigned as the phonon sidebands of P3_BC_, because the energy difference between P3_BC_ and P2_BC_ increases with increasing temperature, and the asymmetric spectral shape remains similar at 80 K and room temperature. Besides the phonon sidebands [30,31,32], there are several other possible causes for the asymmetric PL spectra shape in CsPbBr_3_: (1) At low temperatures (<180 K), a local dipole moment induced by the preferential localization of Cs^+^ in off-center positions of the empty space between the surrounding PbBr_6_ octahedra could generate the Stark effect, generating asymmetric low-temperature PL of CsPbBr_3_; at a higher temperature, around 300 K, this effect should not be important according to the theoretical calculation in Ref. [33]. (2) The existence of an Urbach tail would generate a so-called band-tail effect on photoluminescence, which not only generates an asymmetric PL spectral shape, but also enlarges the energy difference between free excitons emission and band-tail-related recombination because the Urbach tail extends deeper at a higher temperature [34,35,36]. (3) Particularly in 2D CsPbBr_3_ nano-plates, symmetric PL spectra are observed at 77 K [37], and asymmetric PL spectra are observed at 290 K; this asymmetric PL is ascribed to the momentarily trapped excitons–polarons with ~34 meV below the energy level of free excitons [37]. Although exciton–polaron effects had been only extensively discussed in 2D perovskite, the possibility that they caused some asymmetric effects on the PL spectra of 3D perovskite cannot be totally ignored [37]. Therefore, we would like to ascribe the existence of P2_BC_ (or asymmetric PL spectra at higher-photon-energy side in Figure 2a) to multiple factors, including phonon sidebands, band-tail effect, and exciton–polaron effect at high temperature and localization of Cs^+^ in off-center positions at a low temperature. The summary of peak labels and their assignments are included in the Supplementary Information, Table S1. A schematic of the energy diagram describing PL transitions is included in Figure S4. We note that the phonon sideband and band-tail effect could be the dominant mechanism for P2_BC_/P2_F_; however, we cannot rule out the possibilities of contribution from exciton–polaron and the Stark effect caused by Cs^+^ off-centering. Unambiguous separation of these contributions would require time-resolved PL measurements at various temperatures at the sub-picosecond scale, with fine spectral resolution, but such practices are beyond the scope of the present study; however, they will be pursued in future work. Furthermore, we should point out that there would be other components, such as CsPb_2_Br_5_ and Cs_4_PbBr_6_, co-existing within the CsPbBr_3_ crystals, resulting in multi-peaked PL spectra too [38]; on the other hand, both CsPb_2_Br_5_ and Cs_4_PbBr_6_ show much different PL lifetimes compared to CsPbBr_3_ [39,40]. Measured by the time-correlated single-photon counting (TCSPC) technique, the PL dynamics of P1_BC_, P2_BC_, and P3_BC_ at room temperature are shown in Figure S5, their dynamics are only modestly different. Thus, the multi-peaks shown in Figure 2a should not be ascribed to the existence of CsPb_2_Br_5_ and Cs_4_PbBr_6_.

Additional P1_BC_ peaks at 2.21 eV (561 nm) at room temperature and blue-shifts with decreasing temperatures were ascribed to the emission from trapped excitons [19]. Here, we should point out that another possibility at this energy position would be due to the emission from donor–acceptor pairs (DAPs). However, DAPs’ emissions are usually much broader and tend to disappear at high temperatures [19,41], which is not the case in the current work. On the other hand, there is only one peak (named as PTP_BC_) observed to be excited with the fs pulsed laser here. Since the fs laser is at 800 nm, this is from two-photon absorption excitation. The peak excited by 800 nm (PTP_BC_) is almost identical to the P1_BC_ excited by 400 nm CW laser.

Furthermore, Figure 3a shows the temperature dependence of the full width at half maximum (FWHM) of the peaks P1_BC_, P2_BC_, and P3_BC_ excited by the 405 nm CW laser. The values of FWHM were extracted from multiple-peak Lorentz fitting. The typical multi-peak fittings are shown in Figure S3. The FWHM, as a function of temperature, can be described as follows:

(1) $$\Gamma_{0}\left(T\right)=\Gamma_{0}+\gamma_{LO}/({\mathrm{e}}^{\frac{E_{LO}}{k_{B}T}}-1)$$

where Γ_0_ is inhomogeneous broadening without temperature dependence, *γ*_LO_ is the electron–phonon-coupling strength for the Fröhlich interaction, E_LO_ is the averaged energy of the LO phonons involved in optical processes, and k_B_ is the Boltzmann constant. Here, the contribution of acoustic phonons and impurities for broadening PL are ignored, for the impurities will present saturation at a high temperature, and acoustic phonon scattering is more important at a low temperature with linear broadening [42,43].

The E_LO_ for P1_BC_, P2_BC_, and P3_BC_ broadening is 34 meV, 23 meV, and 20 meV, and the γ_LO_ is 85, 62, and 45 meV, respectively. The values are summarized in Table 1. The E_LO_(P3_BC_) and E_LO_(P2_BC_) are roughly the same, being consistent with the assignment as exciton and its derivatives; the E_LO_(P1_BC_) is obviously larger than the others, and this is also consistent with the assignment of trapped excitons’ emission, because the localization enhances the electron–phonon interaction [44,45]. However, we cannot simply assign the emission from PTP_BC_ excited using 800 nm as the emission from the trapped excitons responsible for P1_BC_. Firstly, excitation at 800 nm apparently penetrates deeper into the crystal, thereby avoiding surface defects/impurities. Therefore, in principle, it will reduce, or even eliminate, the photoexcitation caused by surface defects/impurities. Secondly, the higher-energy side of exciton’s emission from inside position of BC could be self-absorbed, resulting in red-shifted spectra.

At the same time, the temperature evolution of the bandgap, Eg(T), in one-oscillator model is as follows [46]:

(2) $$E\left(T\right)=E_{0}+A_{TE}T+A_{\mathrm{EP}}\times \left(\frac{2}{exp(\frac{E_{ave}}{k_{B}T})-1}+1\right)$$

where E_0_ is the un-renormalized bandgap; A_TE_ and A_EP_ are the weight of the thermal expansion and electron–phonon interaction, respectively; and E_ave_ is the averaged optical phonon energy. To lessen the parameters as much as possible, we used averaged phonon energy obtained by the fitting of FWHM using Equation (1), as described previously.

In perovskite, the A_TE_ is known to be positive [47]; reported values were between ~100 and ~300 μeV/K [28,48,49]. In the current work, the values of A_TE_ were fitted as around 200 μeV/K consistently for all peaks (see Table 1). However, the values of A_EP_ are very different. For P1_BC_, A_EP_ is about −160 meV, the largest absolute value of A_EP_ of these separated peaks, which is also consistent with the large γ_LO_ value for P1_BC_. We can see that PTP_BC_ has similar value for its A_EP_ and γ_LO_ with P1_BC_ peak, confirming their same origin, i.e., trapped excitons. A comparison between the existing literature on solution-grown CsPbBr_3_ single crystals and this study is summarized in Table S2, revealing that comparable results can be obtained through analogous testing methods, which also provide support for our identification of the luminescence process. Moreover, in Figure S5, the P1_BC_ presents a modestly longer lifetime, giving another piece of evidence that the P1_BC_ is from the trapped excitons, rather than excitons responsible for P3_BC_. Based on the fitting results shown in Table S3, the modestly longer average lifetime of P1_BC_ (≈13.47 ns) compared with P3_BC_ (≈5.64 ns) further supports its assignment to trapped excitons.

**Figure 4:**
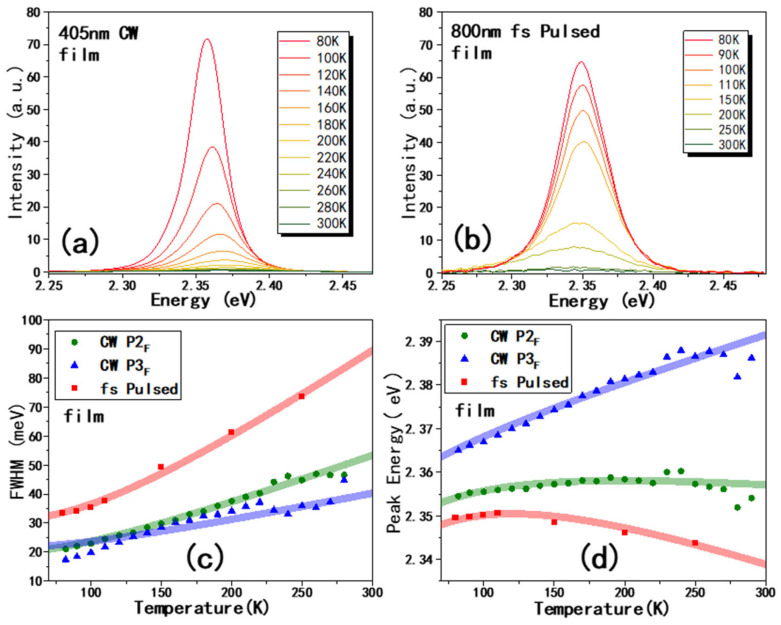
The PL spectra of CsPbBr_3_ films at temperatures ranging from 80 to 300 K, excited by 405 nm CW laser (**a**) and 800 nm fs pulsed laser (**b**). (**c**,**d**) FWHM and peak position excited by 405 nm CW laser (green and blue) and 800 nm fs pulsed laser, respectively. The peak assignments are shown in Table S1 of Supplementary Information.

To test the generality of our previous conclusion, we measured the CsPbBr_3_ thin film. The thickness of the film was estimated as ~300 nm, with the backscattering geometry to collect PL too. In Figure 4a,b, we present the PL of CsPbBr_3_ film from the 405 nm CW laser and 800 nm pulsed laser, respectively. The spectral shape of Figure 4a is not symmetric; thus, it was divided into two peaks using the Lorentz function; the typical fitting results are shown in Figure S6. Compared with peaks energy in PL of BCs, we named them P2_F_ and P3_F_ respectively. The energy difference between P2_F_ and P3_F_ ranges from 12 meV to 31 meV. Therefore, we can assume that P2_F_ contains similar contributions, including a phonon sideband, band-tail effect, etc., similar to the origin of P2_BC_ in Figure 2. We also did not observe an additional P1_BC_-like peak in the film. On the other hand, the PL excited from the 800 nm pulsed laser is also single-peaked, where the peak energy is smaller than P2_F_ (as shown in Figure S7).

In Figure 4c, the FWHM of P2_F_ and P3_F_, as well as PTP_F_, was included. From Equation (1), the E_LO_(P2_F_) and E_LO_(P3_F_) are obtained as 21 and 19 meV, respectively, with γ_LO_(P2_F_) and γ_LO_(P3_F_) as 41 and 39 meV, respectively. The phonon energy and value of γ_LO_ are both smaller than that in bulk crystals, proving smaller electron–phonon interaction in the film.

This is also consistent with the blueshift of peak energy of P3_F_ with much smaller A_EP_ values (also included in Table 1). The peak of PTP_F_ still redshifts with the increasing the temperature. The E_LO_, γ_LO_(PTP_F_), and A_EP_(PTP_F_) are quite different with the value from P2_F_ and P3_F_. Thus, we conclude that the PTP_F_ is also from trapped excitons like the PTP_BC_; the difference is that the trap position is shallower than that in bulk crystals here. Actually, the absence of trapped excitons emission in film using 405 nm excitation may also be due to the shallow energy level of traps, which is overshadowed by P2_F_.

Thus, we readily conclude that the two-photon excited trapped exciton’s emission has a larger electron–phonon interaction. Since the crystal is solution-processed, as shown in XRD, the existence of twin planes proves the existence of structural complexity and instability. The twin planes observed in XRD may be associated with local lattice distortions that act as trapping sites; this hypothesis is supported by the markedly stronger electron–phonon coupling in the bulk crystal but requires further microstructural characterization (e.g., TEM) for definitive confirmation. As shown in Ref. [50], by z-scan measurements, the nonlinear optical responses of CsPb(Cl_0.53_Br_0.47_)_3_ are much more pronounced than those of CsPbCl_3_, because the former one presents larger structural destabilization. On the other hand, defects/traps are usually quenching the PL. Here, on the contrary, trapped excitons’ emission can be clearly observed using two-photon excitation. Thus, we readily conclude that two-photon excitation is more sensitive to defects and structural inhomogeneities within the crystal; this sensitivity allows for the preferential observation of trapped excitons’ emissions [51,52].

As we discussed in the Introduction section, in the literature, the PL spectra of CsPbBr_3_ nanoparticles from TPA and OPA excitation are similar, as both are from the free excitons’ emission. Here, in CsPbBr_3_ BC with ~millimeters size, PL spectra of TPA and OPA are quite different: TPA induced mainly trapped excitons emission, while OPA induced both free excitons and trapped excitons emission. We may deduce that the crystalline size influences the emissions under both TPA- and OPA-excited emission. For quantum dots, the defects mainly exist at the surface, if we assume there are some QDs with the un-passivated surface defects, since the size of QDs is close to the exciton’s size, then excitons are easily quenched by the surface defects: those “defected” QDs cannot emit light at all. Therefore, the PL spectra of TPA- and OPA-induced emissions in nanoparticles are both from free excitons, with almost identical spectra. In the bulk materials, the crystalline grain size will be big enough, and the trap/defect would influence the excitons’ wave function but without quenching its emission. Thus one-photon absorption (OPA) above the bandgap will generate free excitons and trapped excitons’ emission together; on the other hand, third-order nonlinear optical response, which includes TPA, would be much more pronounced by the defect/trap-induced structural destabilization in bulk crystals [50], leaving only trapped exciton emission in the current work. To the best of our knowledge, this is the first report in which one- and two-photon excitation were used together to probe trapped excitons in one piece of solution-grown CsPbBr_3_ bulk crystal, achieving the selective observation of trapped-exciton emission under two-photon excitation and the demonstration of two-photon photoluminescence as a bulk-defect probe. Compared to the other methods, such as thermoluminescence (TSL) and TRPL, as well as theoretical calculation to probe the defects in CsPbBr_3_ [53,54,55,56], TPA is relatively simple and can penetrate to the depth of the bulk crystal, avoiding the surface detects. On the other hand, the method using TPA PL in the current work lacks spatial resolution; thus, it cannot distinguish defect types (such as point or line defects) and their distribution within crystals.

## 4. Conclusions

In summary, we employed a solution-based method to grow CsPbBr_3_ bulk crystal in the current work. Within the temperature range of 80 K to 300 K, excited by a 405 nm CW laser, emissions from excitons and trapped excitons were observed. These emissions generally redshifted with increasing temperature, especially the emission from trapped excitons, which are on the contrary to the bandgap’s relationship with temperature. Analysis of the FWHM proved strong electron–phonon interactions existing in CsPbBr_3_ bulk crystal. Moreover, under two-photon excitation using 800 nm fs pulsed laser, only the emission from trapped excitons was observed in bulk crystal. Because two-photon fluorescence can penetrate deep into the material, it can be used as a method to directly detect the internal quality of the crystal.

## Figures and Tables

**Table 1:** Fitting parameters in Equations (1) and (2). The peak assignments are shown in Table S1 of Supplementary Information.

	Excitation Source	PL Peak	PL Peak Energy at 80 K (eV)	E_LO_ (E_ave_) (meV)	γ_LO_ (meV)	A_TE_ (μeV/K)	A_EP_ (meV)
Bulk crystal	405 nm CW laser	P1_BC_	2.2912	34.16	84.56	211.0	−156.7
		P2_BC_	2.3303	23.58	61.94	200.12	−62.4
		P3_BC_	2.3466	20.11	45.49	210.49	−35.8
	800 nm fs pulsed	PTP_BC_	2.2924	30.76	85.11	211.0	−139.0
Film	405 nm CW laser	P2_F_	2.3546	20.64	40.46	211.54	−27.3
		P3_F_	2.3650	19.39	39.56	200.78	−8.3
	800 nm fs pulsed	PTP_F_	2.3494	25.18	96.32	199.53	−46.5

## Data Availability

The original contributions presented in this study are included in the article. Further inquiries can be directed to the corresponding author.

## Supplementary Materials

The following supporting information can be downloaded at https://www.mdpi.com/article/10.3390/ma19071303/s1, Figure S1. XRD pattern of CsPbBr_3_ film and bulk crystal; Figure S2. The PL spectra of CsPbBr_3_ bulk crystals excited by 405 nm CW laser (a) and 800 nm fs pulsed laser (b) over the temperature range of 80–300 K; Figure S3. The photoluminescence spectra of CsPbBr_3_ bulk crystals at 80 K (a) and 300 K (b). P1_BC_ represents the low-energy peak, while P2_BC_ and P3_BC_ constitute the high-energy peak; Figure S4: Schematic of energy diagram describing PL emission, where “3” is for P3 of free excitons; and “2” is for P2 as phonon sideband (as red ball), band tail effect (as red curve), and other possible mechanisms. A detailed discussed is included in revised manuscript. The “1” is for P1 of trapped excitons; Figure S5. Time-resolved photoluminescence (TRPL) spectra of CsPbBr_3_ bulk crystal at 298 K, with peak separation referenced from Figure 2d. The red, green, and blue curves correspond to the TRPL spectra of the P1_BC_, P2_BC_, and P3_BC_, respectively. (P1_BC_ dynamics is measured at 560 nm, the P3_BC_ is at 520 nm, and the P2_BC_ is at 540 nm.); Figure S6. The photoluminescence spectra of CsPbBr_3_ thin films measured under 405 nm CW laser excitation, two peaks can be resolved, labeled as P2_F_ and P3_F_. The experimental and fitting curves at 80 K (a) and at 250 K (b); Figure S7. The photoluminescence spectra of CsPbBr_3_ thin films excited by 405 nm CW laser (black solid line) and 800 nm fs pulsed laser (red dashed line), compared at 80 K (a) and 250 K (b). The figures below show the normalized temperature-dependent PL spectra excited by 405 nm CW laser (c) and 800 nm fs pulsed laser (d); Table S1. Summary of peak labels and assignments; Table S2. Comparison of our values with those from the most relevant prior single-crystal studies; Table S3. TRPL fitting results and average lifetime [23,28,49,57,58].

## Abbreviations

The following abbreviations are used in this manuscript:

PL	photoluminescence
OPA	one-photon absorption
TPA	two-photon absorption
BC	bulk crystal
CW	continuous wave
XRD	X-ray diffraction
TRPL	time-resolved photoluminescence
TCSPC	time-correlated single-photon counting
DAP	donor–acceptor pairs
