# Supplementary material for: Comparison of One- and Two-Photon Photoluminescence of Solution-Grown CsPbBr3 Bulk Crystals
Source: Materials (Basel). 2026 Mar 25;19(7):1303. doi: 10.3390/ma19071303 (PMC13072991; doi:10.3390/ma19071303)
Supplement: Supplementary file 1 [file materials-19-01303-s001.zip › materials-4180430-supplementary.pdf]

Supporting Information

Comparison of One- and Two-Photon Photoluminescence of Solution-Grown $CsPbBr_3$ Bulk Crystals

Da-Chuan Li [1], Zheng-Da Dong [1], Hou Wang [1], Yang Zhang [2,*] and Chuan-Xiang Sheng [1,*]

1 State Key Laboratory of Photovoltaic Science and Technology, Department of Optical Science and Engineering, School of Information Science and Technology, Fudan University, Shanghai 200433, China
2 School of Electronic Information Engineering, Guangdong University of Petrochemical Technology, Maoming 525000, China
* Correspondence: shuyesuifengqiwu@163.com (Y.Z.); cxsheng@fudan.edu.cn (C.-X.S.)

XRD Patterns

As shown in Figure S1 (part of the Figure 1 in manuscript) following, the peaks are not exactly same for film and for bulk crystals, where peaks for film are at 15.19 (100); for bulk crystals are at 15.31 (002) and 15.45 (110), respectively.

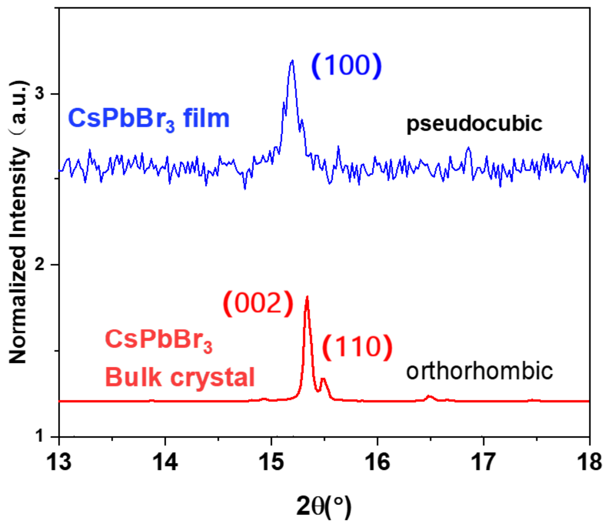

Figure S1. XRD pattern of $CsPbBr_3$ film and bulk crystal.

At the first, we discuss bulk crystals. According to the Bragg equation and $\lambda = 0.154$ nm for Cu Kα, the interplanar distance (d(XRD)) for bulk crystals is d_{002}(XRD) = 0.578 nm and d_{110}(XRD) = 0.573 nm, respectively.

At the same time, for orthorhombic phase of $CsPbBr_3$, there are three lattice constants [1], a=0.819 nm, b = 0.824 nm, c = 1.176 nm (here we note that different source may supply slightly different values, also different growth methods could result in slightly different lattice constant.).

Interplanar spacing of (hkl) lattice plane (d) can be expressed as $1/d^2 = h^2/a^2 + k^2/b^2 + l^2/c^2$.

Obviously, the interplanar spacing of (d_{002} (Cal)) for $CsPbBr_3$ equals 1.176/2 = 0.588 nm, d_{110} (Cal) = $(ab)/((a^2+b^2)^{0.5})$ = 0.581 nm.

Although the d(Cal) is not exactly same with the values from XRD patterns, they are close enough to support labels shown in Figure 1 for bulk crystals. Normally, the phase of $CsPbBr_3$ film was named to be orthorhombic at room temperature is because the single crystal of $CsPbBr_3$ at room temperature is orthorhombic. The cubic phase for $CsPbBr_3$ is with the

lattice constant of 0.587 nm, which is measured at 473 K [1]. On the other hand, the films should exist more crystalline planes, so sometime people used so called pseudo-cubic cell to describe the crystalline structure for film, according to its cubic like XRD patterns [1].

Thus, the XRD peak shown in Figure S1 here can be taken as the (100) plane of pseudo-cubic phase, where the d_{100}(Cal), named as pseudo-cubic lattice constant, is 0.583 nm, being consistent with the lattice constant (0.587 nm) of cubic phase. Actually, we also can name (100) peak of film as ((002) and/or (110)) of orthorhombic phase as well, but since it is not discernable, making not too much sense.

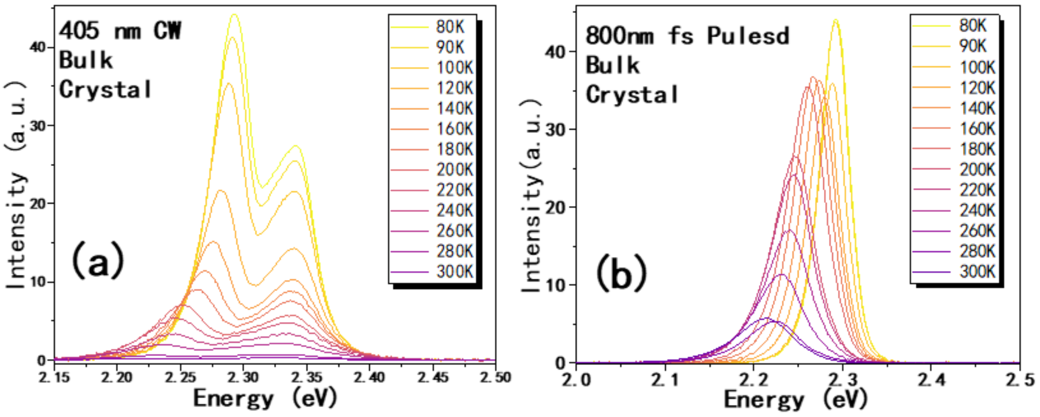

Figure S2. The PL spectra of $CsPbBr_3$ bulk crystals excited by 405 nm CW laser (**a**) and 800 nm fs pulsed laser (**b**) over the temperature range of 80–300 K.

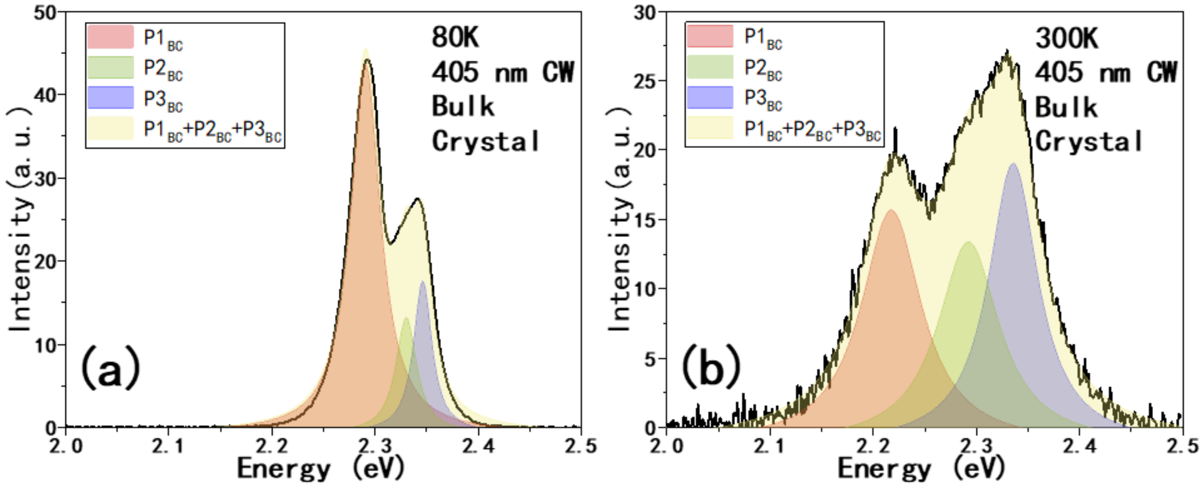

Figure S3. The photoluminescence spectra of $CsPbBr_3$ bulk crystals at 80K (**a**) and 300K (**b**). $P1_{BC}$ represents the low-energy peak, while $P2_{BC}$ and $P3_{BC}$ constitute the high-energy peak. The peak assignments were shown in following Table S1.

Table S1. Summary of peak labels and assignments.

Sample	Excitation	Peak Label	PL Peak Energy at 80 K (eV)	Assignment
Bulk crystal	405 nm CW	$P3_{BC}$	2.3466	Free exciton
	405 nm CW	$P2_{BC}$	2.3303	phonon sideband +band tail *
	405 nm CW	$P1_{BC}$	2.2912	Trapped exciton
	800 nm fs	PTP_{BC}	2.2924	Trapped exciton
Film	405 nm CW	$P3_F$	2.3650	Free exciton
	405 nm CW	$P2_F$	2.3546	phonon sideband +band tail
	800 nm fs	PTP_F	2.3494	Trapped exciton

* However, we cannot rule out the possible involvement of exciton-polaron formation and Stark effect. All figure captions (Figures 2–4) now refer to this Table S1. Peak naming remains consistent (BC = bulk crystal, F = film).

Table S2. Comparison of our values with typical values from literatures for single crystal. FE: Free exciton; TE: trapped exciton.

References	Exciton Species	E_{LO} (meV)	γ_{LO} (meV)	A_{TE} (μeV/K)	A_{EP} (meV)	Trap Depth (meV)	Lifetime (ns)
Solution grown $CsPbBr_3$ crystal [1]	FE	22.2	41.7	140	/	/	/
Solution grown $CsPbBr_3$ crystal [2]	FE	16	46	293	−62	/	/
	TE	20	74	298	−137	30–66 (4–300K)	/
$CsPbBr_3$ film [3]	FE	15	37	92	−35	/	4.02
Solution grown $CsPbBr_3$ Crystal (In this work)	FE	20.11	45.49	210.49	−35.8	/	5.64
	TE	34.16	84.56	211.0	−156.7	54~115 (80–300K)	13.47

[1] Reference [2]; [2] Reference [3]; [3] Reference [4].

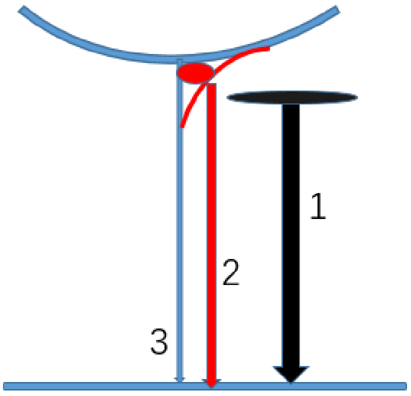

Figure S4. Schematic of energy diagram describing PL emission, where "3" is for P3 of free excitons, "2" is for P2 as phonon side band (as red ball), band tail effect (as red curve), as well as other possible mechanism, detailed discussed was included in revised manuscript. "1" is for P1 of trapped excitons.

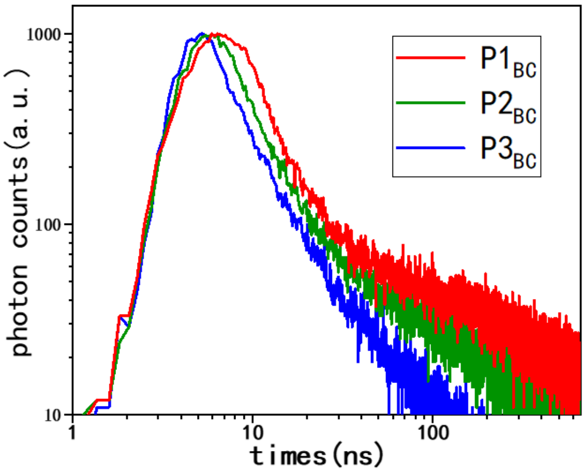

Figure S5. Time-resolved photoluminescence (TRPL) spectra of $CsPbBr_3$ bulk crystal at 298 K, with peak separation referenced from Figure 2d. The red, green, and blue curves correspond to the TRPL spectra of the $P1_{BC}$, $P2_{BC}$, and $P3_{BC}$, respectively. ($P1_{BC}$ dynamics is measured at 560 nm, the $P3_{BC}$ is at 520 nm, and the $P2_{BC}$ is at 540 nm).

According to the literature [5], the biexponential fitting equation (S1) and the average lifetime Equation (S2) were employed to fit curves in Figure S5.

$$I(t) = A_1 e^{\frac{t}{\tau_1}} + A_2 e^{\frac{t}{\tau_2}} + c \tag{S1}$$

$$\tau_{ave} = \frac{A_1\tau_1 + A_2\tau_2}{A_1 + A_2} \tag{S2}$$

Here, $I(t)$ represents the photoluminescence intensity at time t. A_1 and A_2 are the amplitudes (pre-exponential factors) corresponding to the fast and slow decay components, respectively. τ_1 and τ_2 are the corresponding lifetime constants for the fast and slow decay processes. The constant term c accounts for the background offset. The average lifetime τ_{ave} represents the intensity-weighted mean lifetime of the entire decay process. By applying Equation (S1) and Equation (S2) to fit the data in Figure S5, the fitting results are summarized in Table S3.

Table S3. TRPL fitting results and average lifetime.

	A_1	τ_1	A_2	τ_2	τ_{ave}
$P1_{BC}$	1125.4	5.9 ns	51.8	178 ns	13.47 ns
$P2_{BC}$	1019.4	4.1 ns	71.3	70.7 ns	8.45 ns
$P3_{BC}$	984	3 ns	119.4	27.4 ns	5.64 ns

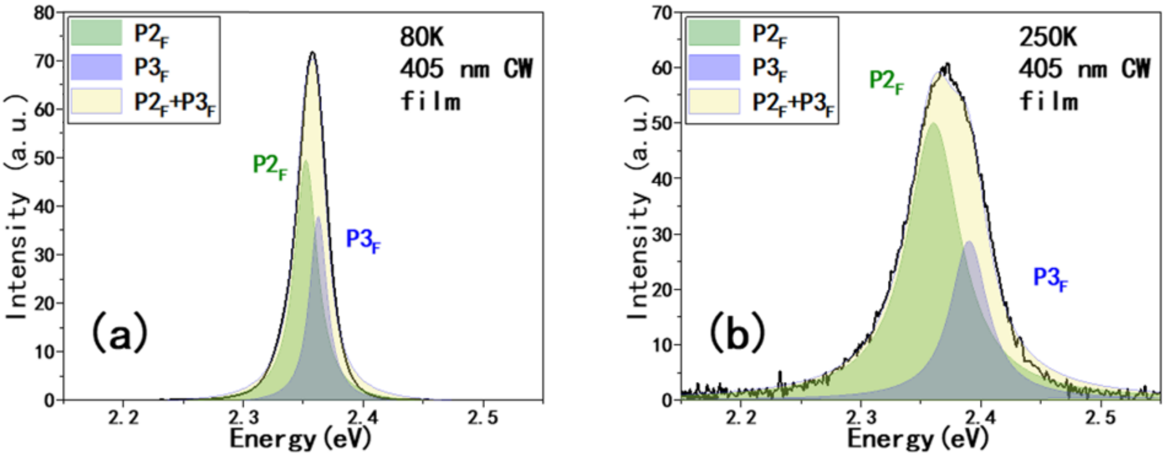

Figure S6. The photoluminescence spectra of $CsPbBr_3$ thin films measured under 405 nm CW laser excitation, two peaks can be resolved, labeled as 2_F and $P3_F$. The experimental and fitting curves at 80 K (**a**) and at 250K (**b**).

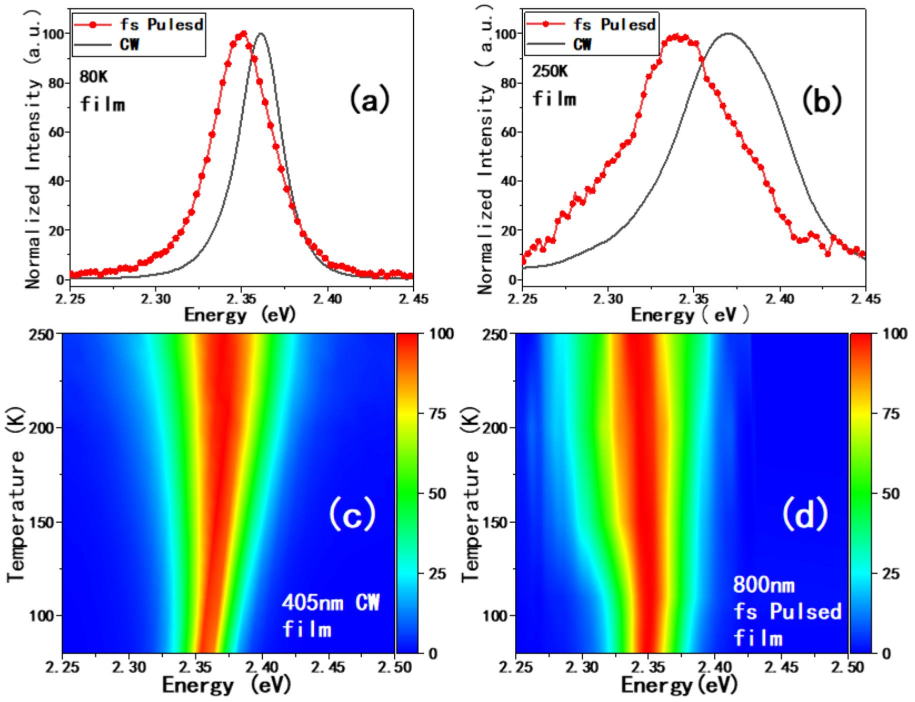

Figure S7. The photoluminescence spectra of $CsPbBr_3$ thin films excited by 405nm CW laser (black solid line) and 800nm fs pulsed laser (red dashed line), compared at 80K (**a**) and 250K (**b**). The figures below show the normalized temperature-dependent PL spectra excited by 405nm CW laser (**c**) and 800nm fs pulsed laser (**d**).

References

1. López, C.A.; Abia, C.; Alvarez-Galván, M.C.; Hong, B.K.; Martínez-Huerta, M.V.; Serrano-Sánchez, F.; Carrascoso, F.; Castellanos-Gómez, A.; Fernández-Díaz, M.T.; Alonso, J.A. Crystal Structure Features of CsPbBr(3) Perovskite Prepared by Mechanochemical Synthesis. *ACS Omega* **2020**, *5*, 5931–5938. https://doi.org/10.1021/acsomega.9b04248.
2. Zhou, X.; Zhang, Z. Electron–phonon coupling in $CsPbBr_3$. *AIP Adv.* **2020**, *10*, 125015.
3. Shibata, K.; Yan, J.; Hazama, Y.; Chen, S.; Akiyama, H. Exciton Localization and Enhancement of the Exciton–LO Phonon Interaction in a $CsPbBr_3$ Single Crystal. *J. Phys. Chem. C* **2020**, *124*, 18257–18263. https://doi.org/10.1021/acs.jpcc.0c06254.
4. Zhang, X.Y.; Pang, G.T.; Xing, G.C.; Chen, R. Temperature dependent optical characteristics of all-inorganic $CsPbBr_3$ nanocrystals film. *Mater. Today Phys.* **2020**, *15*, 100259. https://doi.org/10.1016/j.mtphys.2020.100259.
5. Chen, J.; Lv, J.; Liu, X.; Lin, J.; Chen, X. A study on theoretical models for investigating time-resolved photoluminescence in halide perovskites. *Phys. Chem. Chem. Phys.* **2023**, *25*, 7574–7588. https://doi.org/10.1039/D2CP05723A.

Disclaimer/Publisher's Note: The statements, opinions and data contained in all publications are solely those of the individual author(s) and contributor(s) and not of MDPI and/or the editor(s). MDPI and/or the editor(s) disclaim responsibility for any injury to people or property resulting from any ideas, methods, instructions or products referred to in the content.
